# Clinical Predictors of Locally Advanced Pathology in Esophageal Adenocarcinoma

**DOI:** 10.7759/cureus.18991

**Published:** 2021-10-23

**Authors:** Juan David Gomez Cifuentes, Mahnur Haider, Madhusudhan R Sanaka, Prabhat Kumar, James Bena, John McMichael, Davendra P Sohal, Siva Raja, Sudish Murthy, Prashanthi N Thota

**Affiliations:** 1 Department of Gastroenterology and Hepatology, Baylor College of Medicine, Houston, USA; 2 Section of General Internal Medicine, Tulane Medical Center, New Orleans, USA; 3 Center of Excellence for Barrett’s Esophagus, Department of Gastroenterology and Hepatology, Cleveland Clinic, Cleveland, USA; 4 Department of Quantitative Health Sciences, Cleveland Clinic Foundation, Cleveland, USA; 5 Department of General Surgery, Cleveland Clinic Foundation, Cleveland, USA; 6 Department of Hematology and Oncology, University of Cincinnati, Cincinnati, USA; 7 Department of Thoracic and Cardiovascular Surgery, Cleveland Clinic, Cleveland, USA

**Keywords:** pathology staging, esophageal cancer, neoadjuvant chemotherapy, endoscopic ultrasound, esophageal adenocarcinoma

## Abstract

Background

In patients with resectable esophageal adenocarcinoma (EAC), the decision for neoadjuvant treatment depends on clinical staging with endoscopic ultrasound (EUS) and positron-emission tomography (PET) scan. Patients with locally advanced EAC pathology misclassified as early EAC by clinical staging are missing the opportunity to receive neoadjuvant therapy. We aim to identify predictors of locally advanced pathology in EAC to determine more accurately those who benefit from neoadjuvant therapy.

Methods

Retrospective study of patients who underwent upfront endoscopic or surgical resection for EAC without neoadjuvant therapy from January 2011 to December 2017 was performed. Clinical characteristics, EUS, PET scan and histologic findings were analyzed. Multivariable analysis of predictors of locally advanced stage was performed and a risk prediction score was developed.

Results

A total of 97 patients were included; 68 patients were staged as early EAC (pT1 or pT2 and pN0) and 29 patients were staged as locally advanced EAC (pT1 or pT2 with pN1 and pT3 or pT4 irrespective of N status). In a predictive model of EAC, patients presenting with dysphagia, tumor size >2 cm, exophytic mass appearance on endoscopy and absence of hiatal hernia were more likely to be have locally advanced pathology with a probability of 70% (C-statistic 0.766).

Conclusions

A risk prediction model based on the presence of dysphagia, tumor size >2 cm, exophytic mass appearance and absence of hiatal hernia can be used to identify locally advanced pathology in EAC patients.

## Introduction

Accurate pathologic staging of esophageal adenocarcinoma (EAC) is essential to determine optimal treatment strategy. In patients with localized tumors that are potentially resectable, the decision of treatment modality is highly dependent on clinical staging by endoscopic ultrasound (EUS) and positron-emission tomography (PET) scan: patients with early EAC (T1/T2 with N0) can be managed by upfront resection only, either endoscopic (T1a and selected cases of T1b) or surgical (T1b and T2) [1]; whereas patients with locally advanced EAC (T1/T2 with N positive and T3/T4 irrespective of N status) benefit from neoadjuvant therapy prior to esophagectomy [2,3].

Nonetheless, the endosonographic distinction between T2 tumors that invade until the muscularis propria, and T3 tumors that extend into the adventitia is challenging. In fact, EUS stage concordance with pathologic stage in early EAC has been reported between 30%-53% [4,5]. This distinction is critically important as it determines patients in whom neoadjuvant therapy is indicated. Patients with locally advanced EAC misclassified as early EAC by EUS lose the opportunity to receive neoadjuvant treatment, which maximizes the survival in this group [6].

We hypothesized that certain demographic features, symptoms, PET scan and endoscopic features in EAC patients can help to determine more accurately the locally advanced pathology that benefits from neoadjuvant treatment. Hence, the aim of this study was to identify preoperative predictors of locally advanced pathology in EAC.

## Materials and methods

We conducted a retrospective study of patients who underwent endoscopic or surgical resection for EAC at Cleveland Clinic and other affiliated hospitals from January 1st 2011 to December 31st 2017. Exclusion criteria were: (1) Patients who received any neoadjuvant therapy (to avoid bias from down-staging effects); (2) Patients who had a pathology with undifferentiated carcinoma, neuroendocrine component or no cancer identified on biopsy; (3) Patients in whom EUS staging was incomplete; (4) Patients with PET scan positive for distant metastasis or extraesophageal cancers. Patients were divided by pathologic staging into two groups, early EAC (T1/T2 with N0 on EUS or PET scan) and locally advanced EAC (T1/T2 with N+, and T3/T4 irrespective of N status on EUS or any regional lymph nodes on PET scan). This study was approved on October 26, 2017 by the Institutional Review Board (study number 17-1450) of the Cleveland Clinic, Cleveland, Ohio, United States.

Variables were collected to determine predictors of locally advanced EAC. Variables analyzed include demographic data (age, gender, race), alcohol and tobacco use, body mass index (BMI) at the time of diagnosis, hemoglobin levels within one month of the diagnosis and a prior diagnosis of Barrett’s esophagus. Data regarding the following clinical symptoms at the time of presentation were collected: (1) dysphagia; (2) heartburn and/or regurgitation; (3) post-prandial distress symptoms including early satiety, abdominal fullness, bloating or distention; (4) unintentional weight loss and if present, weight loss in pounds; and (5) gastrointestinal bleeding at presentation. Symptoms were collected through chart review in a binary yes/no fashion.

**Figure 1:**
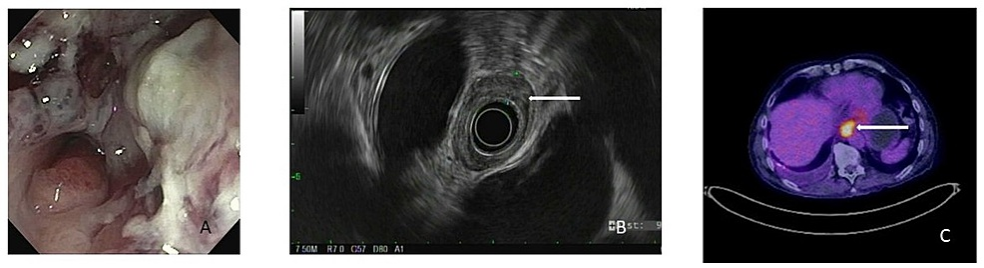
Representative images of esophageal cancer on endoscopy (A), EUS (B) and PET scan (C). EUS: endoscopic ultrasound; PET: positron-emission tomography.

The endoscopic findings abstracted included the presence and length of Barrett’s esophagus, size of hiatal hernia if present, tumor size and circumferential extent of tumor. Tumor endoscopic appearance was divided into four groups: (1) exophytic mass; (2) nodule; (3) submucosal lesion, (4) ulcer. Clinical staging by EUS was compared to pathologic staging; the latter considered the gold standard (Figure 1).

All PET scans were performed following standardized protocol of 12 mCi 18- Fluoro deoxyglucose (FDG) intravenously, followed 1 hour later by PET imaging from the base of the skull to proximal femur. Non-contrast CT was performed for attenuation correction and anatomic localization purposes. PET findings included were standardized uptake value (SUV) of the primary tumor, presence of positive regional nodes and maximal nodal SUV.

Surgical specimens were processed and examined by the Cleveland Clinic Department of Pathology laboratory. Pathologic features included were: tumor histologic type, histologic grade of tumor differentiation, tumor greatest dimension in centimeters, resection margins, lymphovascular and perineural invasion. TNM staging was reported as per the 8th edition of the AJCC/UICC staging manuals for esophageal cancer [7].

Statistical analysis

Categorical variables were described using frequencies and percentages. Continuous variables that were normally distributed were described using means and standard deviations, and continuous variables that were not normally distributed were described using medians and quartiles. Odds ratios, confidence intervals and p-values from logistic regression models predicting advanced stage were calculated. McNemar’s test was used to assess agreement between clinical and pathologic staging.

Multivariable modeling was performed starting with a list of clinically important variables. Logistic regression model with pathologic advanced staging was fitted. In the model, continuous factors were included as restricted cubic splines to allow for non-linear relationships with the outcome. The statistical significance and overall impact of non-linearity terms were evaluated, and where feasible, non-linear terms were removed, or factors were turned into categorical factors. Then, these terms were included in a step-down approach, which uses the predictions from the full model as the outcome, and removes terms that do not affect the prediction of the model; to form a more parsimonious model based on maintaining an explanation of at least 95% of the variability in the full model with the reduced set of predictors. Finally, effects with p-values above 0.10 were removed to make more parsimonious. Odds ratios with 95% confidence intervals are shown, along with bootstrap validated measures of calibration and discrimination. Using the multivariable model fit for advanced pathologic staging, a scoring system was created using the method described by Sullivan et al. [8]. Briefly, regression coefficients from the final multivariable model were divided by the smallest coefficient and rounded to the nearest integer. These scores were then summed to form the risk score. The estimated risk of advanced staging was calculated as the predicted probability from the model with the intercept and risk score as entries. The discrimination of the model based on the risk score was also calculated. Analyses were performed using SAS® Software (version 9.4; Cary, NC) and R software (version 3.6; Vienna, Austria).

## Results

**Figure 2:**
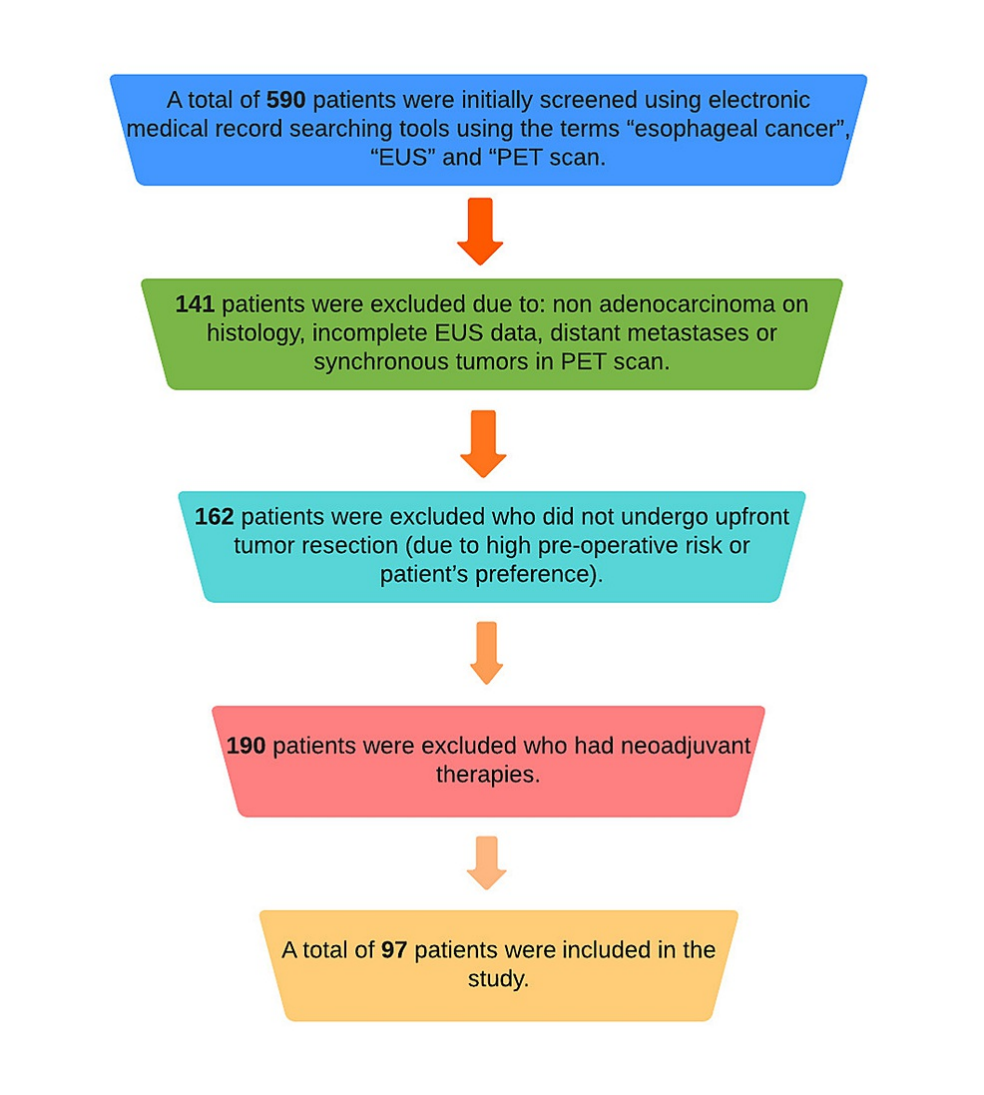
Flowchart of screening the patients. EUS: endoscopic ultrasound; PET: positron-emission tomography.

A total of 590 patients were initially screened using electronic medical record searching tools using the terms “esophageal cancer”, “EUS” and “PET scan”; 141 patients were excluded due to: nonadenocarcinoma on histology, incomplete EUS data, distant metastases or synchronous tumors in PET scan. Other patients excluded were 162 who did not undergo upfront tumor resection (due to high pre-operative risk or patient’s preference) and 190 who had neoadjuvant therapies. Finally, a total of 97 patients were included in the study (Figure 2).

Based on histologic examination, pathologic staging was early EAC (T1 or T2 and N0) in 68 (70.1%) patients and locally advanced EAC (T1/T2 with N positive and any T3/T4) in 29 (29.9%) patients. In the early EAC group the staging distribution was as follows: pT1aN0=33 (48.5%), pT1bN0=29 (42.6%) and pT2N0=6(8.9%). In the locally advanced EAC group, the distribution was: pT1aN+=1 (3.4%), pT1bN+=4 (13.8%), pT2N+=3 (10.3%), pT3N0=5 (17.2%), pT3N+=13 (44.95) and pT4aN+=3 (10.3%). In the early EAC group, 44 patients (64.7%) underwent esophagectomy and 24 (35.3%) underwent endoscopic resection, whereas all patients with locally advanced EAC underwent esophagectomy. None of the patients underwent any neoadjuvant therapy. 

Demographic and clinical features

Among the cohort, the average age was 65.8 ± 9.6 years, 85.6% of the patients were male and 93.8% of Caucasian race (Table1). When comparing groups, there were no significant differences between the groups in terms of age, gender, race, and variables evaluating alcohol and tobacco use. Patients staged with locally advanced cancer had lower BMI at the time of diagnosis (28.8 ± 6.4 vs. 22.8 ± 4.4 p=<0.001) than patients staged as early EAC. In symptom presentation, more patients in the locally advanced EAC group presented with dysphagia (58.6% vs 30.9%, p=0.010) and weight loss (37.9% vs 17.6 %, p=0.032). The prevalence of other symptoms and hemoglobin level at initial presentation were comparable (Table 1).

**Table 1:** Patient characteristics. Statistics presented as mean ± SD, median [P25, P75], N (column %). p-values: ^a^t-test, ^b^Wilcoxon rank sum test, ^c^Pearson's chi-square test, ^d^Satterthwaite t-test. BMI: body mass index; GI: gastrointestinal.

Factor	Overall (N=97)	Early stage (N=68)	Advanced stage (N=29)	p-value
Age at diagnosis (years)	65.8 ± 9.6	66.8 ± 9.4	63.3 ± 9.9	0.11^a^
Male Gender	83 (85.6)	57 (83.8)	26 (89.7)	0.45^c^
White race	91 (93.8)	64 (94.1)	27 (93.1)	0.85^c^
Current/former smoker	70 (72.2)	48 (70.6)	22 (75.9)	0.60^c^
If smoker; pack years	25.1 ± 14.4	25.5 ± 14.8	24.1 ± 14.0	0.73^a^
History of alcohol use	5 (8.5)	2 (5.0)	3 (15.8)	0.16^c^
Never/quit alcohol	12 (12.4)	9 (13.2)	3 (10.3)	0.77^c^
BMI (kg/m^2^) at diagnosis	27.0 ± 6.5	28.8 ± 6.4	22.8 ± 4.4	<0.001^d^
Hemoglobin (gm/dL)	13.8 ± 1.9	13.6 ± 2.0	14.2 ± 1.7	0.25^a^
Dysphagia	38 (39.2)	21 (30.9)	17 (58.6)	0.010^c^
Odynophagia	1 (1.03)	1 (1.5)	0 (0.00)	0.51^c^
Heartburn/regurgitation	36 (37.1)	24 (35.3)	12 (41.4)	0.57^c^
Chest pain	7 (7.2)	5 (7.4)	2 (6.9)	0.94^c^
Post prandial distress	10 (10.3)	7 (10.3)	3 (10.3)	0.99^c^
Unintentional weight loss	23 (23.7)	12 (17.6)	11 (37.9)	0.032^c^
Weight loss (lbs.)	15.0 [10.0, 26.0]	24.5 [10.0, 26.0]	10.0 [10.0, 15.0]	0.44^b^
GI bleeding at presentation	19 (19.6)	15 (22.1)	4 (13.8)	0.35^c^
Prior diagnosis of Barrett’s esophagus	68 (70.1)	51 (75.0)	17 (58.6)	0.11^c^

Endoscopic findings

On the endoscopy, most tumors were in the distal esophagus; the mean location of the gastroesophageal junction in the cohort was 38.6 ± 2.8 cm, the median proximal tumor end was 36.0 ± 3.0cm and the distal tumor end was 38.3 ± 4.0 cm from the incisors. Endoscopic characteristics revealed that patients staged as locally advanced EAC had tumors larger in size (3.0 cm [2.5, 4.0] vs. 2.0 cm [1.40, 3.0]; p=0.005) with a tumor circumference > two-thirds of the lumen (19.2% vs. 1.6%; p=0.002), and more often described as exophytic mass (69.0% vs 38.2%; p=0.002) (Table 2). The presence of Barrett’s esophagus and Barrett’s segment length were similar between the groups (Table 2).

**Table 2:** Endoscopic/PET scan/histologic characteristics by staging group. Statistics presented as mean ± SD, median [P25, P75], N (column %). p-values: ^a^t-test, ^b^Wilcoxon rank sum test, ^c^Pearson's chi-square test, ^d^Satterthwaite t-test. GEJ: gastroesophageal junction; PET: positron-emission tomography; SUV: standardized uptake value.

Factor	Overall (N=97)	Early stage (N=68)	Advanced stage (N=29)	p-value
GEJ location (cm)	38.6 ± 2.8	38.4 ± 3.2	39.1 ± 1.8	0.18^d^
Hiatal hernia presence	45 (46.4)	38 (55.9)	7 (24.1)	0.004^c^
Hiatal hernia (cm)	3.0 [2.0, 4.0]	3.0 [2.0, 4.0]	3.0 [2.0, 3.0]	0.67^b^
Stricture	6 (6.2)	1 (1.5)	5 (17.2)	0.003^c^
Barret’s Esophagus presence	46 (49.5)	36 (56.3)	10 (34.5)	0.052^c^
Barret's segment length (cm)	0.00 [0.00, 5.0]	2.5 [0.00, 5.0]	0.00 [0.00, 4.0]	0.080^b^
Tumor size (cm)	2.5 [2.0, 4.0]	2.0 [1.4, 3.0]	3.0 [2.5, 4.0]	0.005^b^
Tumor size >2cm	74 (76.3)	47 (69.1)	27 (93.1)	0.011^c^
Tumor appearance				
Exophytic mass	46 (47.4)	26 (38.2)	20 (69.0)	0.002^c^
Nodule	42 (43.3)	37 (54.4)	5 (17.2)	
Submucosal	4 (4.1)	1 (1.5)	3 (10.3)	
Ulcer	5 (5.2)	4 (5.9)	1 (3.4)	
Tumor circumference				
0-33%	67 (75.3)	53 (84.1)	14 (53.8)	0.002^b^
34-66%	16 (18.0)	9 (14.3)	7 (26.9)	
67-100%	6 (6.7)	1 (1.6)	5 (19.2)	
PET scan findings				
SUV of primary tumor	4.4 [3.0, 6.8]	4.0 [2.9, 6.1]	5.6 [4.5, 9.0]	0.003^b^
SUV of primary tumor				
0-2.5	17 (17.5)	15 (22.1)	2 (6.9)	0.006^b^
2.6-5	43 (44.3)	33 (48.5)	10 (34.5)	
>5	37 (38.1)	20 (29.4)	17 (58.6)	
SUV of primary tumor				
5.0 or less	60 (61.9)	48 (70.6)	12 (41.4)	0.007^c^
Over 5.0	37 (38.1)	20 (29.4)	17 (58.6)	
Nodes				
Negative	78 (80.4)	55 (80.9)	23 (79.3)	0.86^c^
Regional Positive	19 (19.6)	13 (19.1)	6 (20.7)	
SUV of lymph nodes	0.00 [0.00, 0.00]	0.00 [0.00, 0.00]	0.00 [0.00, 0.00]	0.96^b^
SUV of lymph nodes				
0-2.5	84 (86.6)	59 (86.8)	25 (86.2)	0.98^b^
2.6-5	9 (9.3)	5 (7.4)	4 (13.8)	
>5	4 (4.1)	4 (5.9)	0 (0.00)	
Pathology findings of resection specimens:				
Histologic grade				
Well differentiated	17 (18.3)	16 (25.0)	1 (3.4)	0.012^c^
Moderately differentiated	44 (47.3)	31 (48.4)	13 (44.8)	
Poorly differentiated	32 (34.4)	17 (26.6)	15 (51.7)	
Tumor greatest dimension (cm)	2.0 [0.80, 3.4]	1.3 [0.70, 2.5]	3.0 [2.5, 4.2]	<0.001^b^
Positive resection margins	11 (11.3)	8 (11.8)	3 (10.3)	0.84^c^
Lymphovascular invasion	35 (37.6)	14 (21.5)	21 (75.0)	<0.001^c^
Perineural invasion	22 (25.9)	5 (8.3)	17 (68.0)	<0.001^c^

PET scan findings

Regarding PET scan findings, patients with locally advanced EAC had higher median primary SUV (5.6 [4.5, 9.0] vs. 4.0 [2.9, 6.1] p=0.003) and more percentage of tumors with SUV >5 (58.6% vs 29.4% p=0.06) than patients staged as early EAC (Table 2).

Pathology findings of resection specimens

The pathology characteristics revealed that patients staged as locally advanced cancer had larger tumors (3.0 [2.5, 4.2] vs. 1.3 [0.70, 2.5] p=<0.001), poorly differentiated (51.7% vs. 26.6% p=0.012) and with positive lymphovascular (75% vs. 21.5% p=<0.001) and perineural (68% vs. 8.3% p=<0.001) invasion. The rate of positive resection margins did not differ between the groups (10.3% vs. 11.8% p=0.84) (Table 2).

Univariate analysis

In univariate analysis, patients who presented initially with dysphagia (OR 3.2 [1.3, 7.8] p=0.012) and unintentional weight loss (OR 2.9 [1.08, 7.6] p=0.035) were more likely to be staged as locally advanced EAC. A tumor size >2 cm (OR 6.0 [1.3, 27.7] p=0.021), exophytic mass appearance (OR 3.6 [1.4, 9.1] p=0.007), presence of stricture (OR 14.0 [1.6, 125.6] p=0.019), and primary SUV >5.0 on PET scan (OR 6.4 [1.3, 31.9] p=0.024) were the strongest predictors of locally advanced EAC. On the other hand, patients who presented at the time of diagnosis with higher BMI (OR 0.78 [0.69, 0.89] p=<0.001), or had hiatal hernia on endoscopy (OR 0.25 [0.09, 0.67] p=0.006) had a higher likelihood of being staged as early EAC (Table 3).

**Table 3:** Univariable predictors of locally advanced pathology. Statistics presented as mean ± SD, median [P25, P75], N (column %). Odds ratio (OR), confidence intervals (CI) and p-values correspond to univariate logistic regression models. BMI: body mass index; GI: gastrointestinal; GEJ: gastroesophageal junction; SUV: standardized uptake value.

Factor	Odds ratios (95% CI)	p-value
Age (years)	0.96 (0.92, 1.01)	0.11
Male vs female	0.60 (0.15, 2.3)	0.46
Current or former smoker	1.3 (0.48, 3.6)	0.60
BMI at contact date	0.78 (0.69, 0.89)	<0.001
Initial hemoglobin	1.2 (0.89, 1.6)	0.25
Dysphagia	3.2 (1.3, 7.8)	0.012
Heartburn/regurgitation	1.3 (0.53, 3.2)	0.57
Chest pain	0.93 (0.17, 5.1)	0.94
Post prandial distress	1.01 (0.24, 4.2)	0.99
Unintentional Weight loss	2.9 (1.08, 7.6)	0.035
GI bleeding at presentation	0.57 (0.17, 1.9)	0.35
Prior reported Barret’s	0.47 (0.19, 1.2)	0.11
GEJ location cm	1.1 (0.93, 1.3)	0.27
Hiatal hernia	0.25 (0.09, 0.67)	0.006
Stricture	14.0 (1.6, 125.6)	0.019
Barret's length	0.91 (0.80, 1.04)	0.18
Tumor size (cm)	1.3 (1.01, 1.6)	0.042
Tumor >2 cm vs <2 cm	6.0 (1.3, 27.7)	0.021
Tumor appearance: mass	3.6 (1.4, 9.1)	0.007
Primary SUV	1.09 (1.01, 1.2)	0.028
Primary SUV 0-2.5 vs 2.6-5	2.3 (0.44, 11.7)	0.33
Primary SUV 0-2.5 vs >5	6.4 (1.3, 31.9)	0.024
Primary SUV <5.0 vs >5.0	3.4 (1.4, 8.4)	0.008

Multivariable analysis

In multivariable analysis, dysphagia (OR 3.04 [1.12, 8.29] p=0.029), tumor size >2 cm (OR 4.58 [0.92, 22.81] p=0.063), and tumor appearance as exophytic mass (2.84 [1.03, 7.85] p=0.044) were identified as predictors for locally advanced EAC. Conversely, hiatal hernia on endoscopy (OR 0.29 [0.10, 0.83] p=<0.021) increased the likelihood of being classified as early EAC (Table 4).

**Table 4:** Multivariable model predicting pathology staging.

Factor	Level	Odds ratio (95% CI)	p-value	Coefficient	Score
Dysphagia	Yes vs. No	3.04 (1.12, 8.29)	0.029	1.113	1
Hiatal hernia	No vs. Yes	3.43 (1.20, 9.81)	0.021	1.233	1
Tumor size: 2cm+	Yes vs. No	4.58 (0.92,22.81)	0.063	1.522	1.5
Tumor appearance: exophytic mass	Yes vs. No	2.84 (1.03, 7.85)	0.044	1.043	1

Risk prediction score

**Figure 3:**
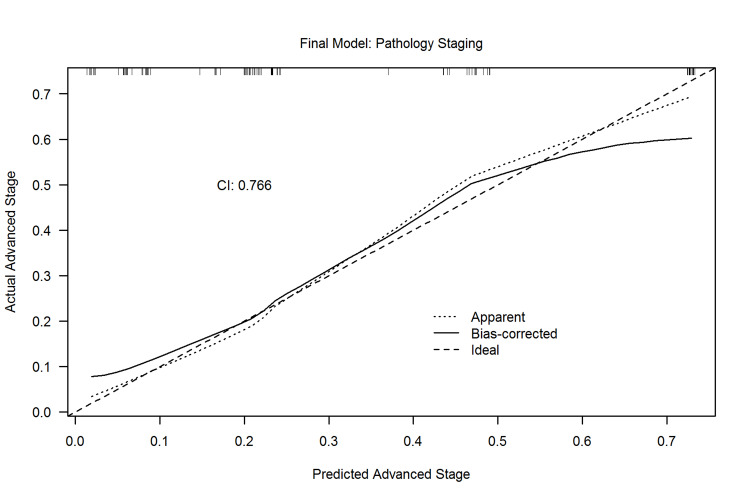
Calibration plot for model to predict pathology staging.

A model was built to predict locally advanced EAC pathologic staging using the factors identified by multivariable analysis (table 5). The calibration plot for the model demonstrates good agreement between predicted and actual risk, especially in the mid-range of the risk, where most of the data exists (Figure 3). The bias-corrected discrimination measure [concordance statistic or index (CI)] was 0.766. A value of 1 means that the model perfectly predicts outcome, values over 0.8 indicate a strong model, values over 0.7 indicate a good model and values below 0.5 indicate a poor model. For example, based on Table 5, a patient presenting with dysphagia and with endoscopic findings of a exophytic mass >2cm in the absence of hiatal hernia has a total score of 5 points that translates into a predicted probability of locally advanced EAC value of 0.75.

**Table 5:** Risk prediction score.

Total score	Estimated risk	Actual risk
0	0.019	0/7 (0%)
1-1.5	0.067	2/19 (10.5%)
2-3	0.173	8/46 (17.4%)
>3	0.603	20/35 (57.1%)

Correlation between clinical and pathologic staging

For clinical vs pathologic staging of the patients with locally advanced EAC, 17/29 (58.6%) were correctly classified and 12/29 (41.4%) patients were incorrectly classified as early EAC by EUS. In contrast, in the early EAC group, 45/68 of patients (66.2%) were classified as such by EUS and 23/68 (33.8%) were in reality locally advanced EAC by pathology. Overall, in 23.7% of cases, the EUS overstaged than pathology, while in 12.5%, EUS understaged pathology stage. Based on the calculated McNemar’s test for agreement (p=0.063), there was not enough evidence to indicate that EUS significantly overstaged the results.

## Discussion

Pathologic staging remains the main prognostic factor for disease-specific survival in esophageal cancer with progressively decreasing survival rates with increasing tumor depth, presence of lymph nodes and increasing number of involved lymph nodes [9]. Therefore, accurate staging is essential to deliver optimal care to maximize survival. We identified four predictors of locally advanced pathology in EAC, namely dysphagia, presence of exophytic mass in the esophagus, tumor size > 2cm and absence of hiatal hernia. We developed a scoring system based on these predictors to identify locally advanced EAC with about 70% probability.

The comparison of clinical and pathologic staging in our cohort revealed that EUS accurately predicted the pathologic T-stage and N stage accurately in approximately 64% of patients. More importantly, 12.5% of patients were understaged by EUS. Preoperative EUS staging in EAC has been studied extensively and although the accuracy of T stage determination is inadequate in early EAC and increases with deeper tumor penetration [10-14]. In a national cancer database study of 1120 EAC patients with clinical T1 and T2 with N0 who underwent esophagectomy without neoadjuvant therapy, pathologic upstaging occurred in 21.3% of patients [15]. Increasing tumor size (tumor size 1-3 cm, OR 4.57, tumor size >3 cm, OR 10.57, as compared to tumors <1 cm), positive margins (OR 4.13) and > than 10 lymph nodes examined (OR 1.85) were associated with upstaging.15 In another study of 2775 patients with EAC, most patients presented with cN0 (82.8%) and cT1 tumors (53.6%) [16]. The overall concordance between clinical and pathologic staging was 78.8% for T‐classification and 78.8% for N‐classification. Patients that were upstaged due to a lack of concordance between T‐classification had decreased 5‐ and 10‐year overall survival (30%and 16%, p < 0.001) and those upstaged due to discordant N‐classification had decreased 5‐ and 10‐year OS (28% and 23%, p < 0.001) as compared to patients who had concordance between clinical and pathological T‐ and N‐classification (5‐year: 54% and 59%; 10‐year:38% and 41%, respectively). This highlights the fact that a percentage of EAC patients are clinically understaged and therefore not offered neoadjuvant therapy which can prolong the survival.

There are several studies reporting on the predictors of unsuspected advanced pathology in clinically early-stage EAC patients. In a Surveillance, Epidemiology, and End Results database study from 2004 to 2010 for patients with early-stage EAC, the predictors of lymph node metastases were tumor grade (odds ratio [OR], 2.76; [95% CI], 1.58-4.82 [p<0.001]), T classification (T1a vs. T1b OR, 0.47; 95% CI, 0.24-0.91 [p =0.025]), and tumor size (OR, 2.68; 95% CI, 1.48-4.85 [p = 0.001]) [17]. For patients with low-grade (well or moderately differentiated) tumors measuring <2 cm in size, the risk of lymph node metastasis was 1.7% for T1a (P<.001) and 8.6% for T1b (p = 0.001) tumors. In a national cancer database study, independent predictors of lymph node metastases were submucosal invasion, lymphovascular invasion (LVI), decreasing differentiation, and tumor size ≥ 2 cm (p < 0.05). For T1a tumors with poor differentiation or size ≥ 2 cm, lymph node metastases rates were 10.2 and 6.7%, respectively. The lymph node metastases rate in well-differentiated T1b tumors < 2 cm was 4.2% [18].

Similarly, another study identified that tumor size >3 cm, higher histologic grade and lymphovascular invasion were key variables, and the presence of any of those was associated with >48.1% risk of pathologic upstaging [19]. It is noteworthy that information about some of the predictors is available only after esophagectomy and therefore not useful for initial treatment decisions.

Measurement of SUV, which reflects the metabolic activity of the tumor, on PET/CT may serve as a prognostic factor. Markedly intense SUV has been documented as predictor for tumor pathology upstaging in clinical early-stage EAC patients (OR 5.76 [1.25, 26.52] p=0.021) [20]. In addition, primary max SUV greater than 2.5 has been associated with positive nodal disease and therefore locally advanced EAC [21].

Dysphagia is a well-established predictor for T3 or T4 disease. In 111 patients with nonmetastatic EAC, the sensitivity, specificity, and positive predictive value of dysphagia grade ≥3 (can only swallow liquids or total dysphagia) for T3 lesions were 36% (95% CI 25-48%), 100% (95% CI 89-100%), and 100% (95% CI 83-100%), respectively [22]. Overall, there was a significant positive correlation between dysphagia grade and the EUS T-stage of esophageal cancer. In another study of 216 patients with EAC [23], Sensitivity and specificity for the presence of dysphagia at the time of esophageal cancer diagnosis in predicting locally advanced disease were 0.83 (95% CI 0.70-0.92) and 0.84 (95% CI 0.78-0.89), respectively. In another prospective study of 114 patients with EAC, among patients with dysphagia, 89% (54 of 61) had T3-4 disease by EUS; among those without dysphagia, only 53% (28 of 53) had T3-4 disease by EUS (p < 0.001) [24]. In a study by Portale et al, patients without dysphagia, and with tumor length <2 cm occupying less than 25% circumference had 82% positive predictive value to be staged as early EAC [25]. However, it is worthwhile to note that these studies examined the association of dysphagia with clinical-stage but not pathologic stage. An unexpected finding in our study is the higher prevalence of hiatal hernia in early-stage EAC (55.9% vs 24.1% p=0.004), and the difference remained significant even after multivariable analysis (p=0.021). It is a well-known fact that hiatal hernia is a risk factor for EAC. In fact, it has been associated with an increased risk of Barrett’s esophagus and neoplastic progression in Barrett’s esophagus [26]. There is also incremental risk with the increasing size of hiatal hernia reported in several studies [27]. One possibility for our study finding may be that patients with large hiatal hernias have poorly controlled acid reflux symptoms [28] and may get an upper endoscopy performed more frequently leading to an earlier detection of EAC.

Several models have been developed for esophageal cancer to identify patients with unsuspected lymph node metastases. In a recent national cancer database study, 688/3186 (22%) of clinical N0 patients who underwent upfront esophagectomy had pathologic lymph node involvement [29]. Variables associated with pN+ status included histology [adenocarcinoma vs squamous: OR 1.75], tumor stage (T1: reference, T2: OR 1.90, T3: OR 2.17), tumor size (<1 cm: reference, 1-2 cm: OR 2.25, 2-3 cm: OR 3.82, 3-4 cm: OR 5.40, 4-5 cm: OR 5.66, ≥5 cm: OR 6.02), grade (1: reference, 2: OR 2.62, 3: OR 4.39, 4: OR 4.15, unknown: OR 1.87), and presence of lymphovascular invasion (absent: reference, present: OR 4.70, missing: OR 1.87), all P < 0.001. A nomogram with these variables had good predictive accuracy (Brier score: 0.14, calibration slope: 0.97, c-index: 0.77). In another study of 258 patients T1 EAC who underwent upfront esophagectomy, a scoring system was developed using tumor size (+1 point per cm), depth of invasion (+2 for T1b), differentiation (+3 for each step of dedifferentiation), and lymphovascular invasion (+6 if present) [30]. With a score of 0 to 1 point, prevalence of lymph node metastasis was ≤2%; with 2 to 4 points, the prevalence was 3% to 6%; and with 5+ points the prevalence was ≥7%. These nomograms rely on information obtained by histopathologic examination such as tumor grade, depth of invasion, and lymphovascular invasion and therefore, are of no utility prior to treatment initiation.

Although some of the clinical predictors identified in our study population have been reported before, the main strength of the study is the development of a risk score for locally advanced pathology in EAC using simple features which are not subject to inter-observer variation. Moreover, this risk score can be used prior to initiating any therapy in patients with resectable EAC. Another strength of our study is the exploration of numerous variables seldom reported in the EAC literature such as upper GI symptoms other than dysphagia, hemoglobin levels, BMI, tumor appearance, and hiatal hernia.

This study has several limitations. Since pathologic staging is available only after esophagectomy, it does not aid in initial treatment planning. However, it is an important prognostic factor for overall survival. The small size of the cohort allowed classification into two groups only, with a small number of patients with T2N0 disease. In addition, this is a retrospective review and the description of tumor appearance was not standardized, increasing the subjectivity of the reports. This has special importance given one of the strongest predictors of locally advanced EAC was the description of “mass” vs “nodule, ulcer or submucosal lesion”; Similarly, the oncologist decision making of why patients clinically staged as locally advanced EAC did not receive neoadjuvant therapies is limited by this same factor. Third, the risk score includes the absence of hiatal hernia, which is a less useful predictor; since it is unlikely to be related to the progression from early to advanced EAC, and probably reflects the fact that patients with hiatal hernia are more likely to be symptomatic and therefore have an early endoscopy. We did not consider tumor grade as one of the prognostic factors as it is not available on all forceps biopsy specimens prior to resection. Although tumor grading is considered in early staging, it is not taken into consideration for stages past IIb [7]. In addition, a small number of patients underwent endoscopic resection. In these patients, true pN status is not known.

## Conclusions

In conclusion, in patients with resectable non-metastatic EAC, dysphagia, tumor size >2 cm and exophytic mass appearance on endoscopy are predictors of locally advanced pathologic stage. Interestingly, the presence of hiatal hernia was an indirect marker of early EAC. These four factors predict 70% probability of advanced pathologic stage. Our study identified exploratory variables that can be used to develop stronger models in larger populations, which could hopefully improve diagnostic accuracy, minimizing the number of patients with locally advanced EAC understaged by clinical staging.
